# Habitat restoration opportunities, climatic niche contraction, and conservation biogeography in California's San Joaquin Desert

**DOI:** 10.1371/journal.pone.0210766

**Published:** 2019-01-15

**Authors:** Joseph A. E. Stewart, H. Scott Butterfield, Jonathan Q. Richmond, David J. Germano, Michael F. Westphal, Erin N. Tennant, Barry Sinervo

**Affiliations:** 1 Department of Ecology and Evolutionary Biology, University of California Santa Cruz, Santa Cruz, CA, United States of America; 2 Institute for the Study of Ecological and Evolutionary Climate Impacts, University of California, Santa Cruz, CA, United States of America; 3 The Nature Conservancy, San Francisco, CA, United States of America; 4 U.S. Geological Survey, San Diego, CA, United States of America; 5 Department of Biology, California State University Bakersfield, Bakersfield, CA, United States of America; 6 U.S. Bureau of Land Management, Marina, CA, United States of America; 7 Lands Unit, Central Region, California Department of Fish and Wildlife, Fresno, CA, United States of America; San Diego Zoo Institute for Conservation Research, UNITED STATES

## Abstract

A recent global trend toward retirement of farmland presents opportunities to reclaim habitat for threatened and endangered species. We examine habitat restoration opportunities in one of the world’s most converted landscapes, California’s San Joaquin Desert (SJD). Despite the presence of 35 threatened and endangered species, agricultural expansion continues to drive habitat loss in the SJD, even as marginal farmland is retired. Over the next decades a combination of factors, including salinization, climate change, and historical groundwater overdraft, are projected to lead to the retirement of more than 2,000 km^2^ of farmland in the SJD. To promote strategic habitat protection and restoration, we conducted a quantitative assessment of habitat loss and fragmentation, habitat suitability, climatic niche stability, climate change impacts, habitat protection, and reintroduction opportunities for an umbrella species of the SJD, the endangered blunt-nosed leopard lizard (*Gambelia sila*). We use our suitability models, in conjunction with modern and historical land use maps, to estimate the historical and modern rate of habitat loss to development. The estimated amount of habitat lost since the species became protected under endangered species law in 1967 is greater than the total amount of habitat currently protected through public ownership and conservation easement. We document climatic niche contraction and associated range contraction away from the more mesic margins of the species’ historical distribution, driven by the anthropogenic introduction of exotic grasses and forbs. The impact of exotic species on *G*. *sila* range dynamics appears to be still unfolding. Finally, we use NASA fallowed area maps to identify 610 km^2^ of fallowed or retired agricultural land with high potential to again serve as habitat. We discuss conservation strategies in light of the potential for habitat restoration and multiple drivers of ongoing and historical habitat loss.

## Introduction

Habitat loss resulting from agricultural expansion is one of the greatest historical drivers of extinction [1,2]. A recent global trend toward retirement of marginal farmland, especially in temperate latitudes, presents an important opportunity to reclaim some of this lost habitat in a cost-effective manner before the land is claimed for other uses [3–6]. More information on patterns of habitat loss, degradation, and use will allow targeted habitat restoration in areas where it will make the biggest impact for conserving sensitive and endangered species.

To better understand this opportunity, we investigate habitat and land use trends in the San Joaquin Desert (SJD) of California [7]. Now largely converted to agriculture, the SJD was once an extensive network of upland and aquatic habitats, supporting a high concentration of endemic species [8]. As a result of habitat loss, 7 species have been driven to extinction or regional extirpation from the SJD, and 35 species are now protected under endangered species legislation (S1 Table). Over the next 30 years a combination of factors, including salinization, climate change, and historical groundwater overdraft, are projected to lead to the retirement of more than 2,000 km^2^ of SJD farmland [9]. If retired farmland can be restored to habitat in strategically selected areas of the SJD, it could contribute to the recovery of dozens of vulnerable species.

While we center our discussion primarily around restoration opportunity, which has great potential to benefit many endangered species, we also present documentation of a rarely documented phenomenon: climatic niche contraction mediated by invasive species. Previous studies documented negative impacts of exotic grasses and forbs on species demography [10,11]. Here we document climatic niche contraction and associated range contraction away from the mesic margin of a species historical distribution. The contraction was driven by the interaction of precipitation and invasive grasses and forbs. Though we suspect the phenomenon is widespread, this study appears to be one of few empirical examples of climatic niche contraction caused by an invasive species (but see [12]). The phenomenon has interesting implications for community reshuffling in the context of climate change.

In this study we focus on habitat change in an iconic endangered species of the SJD, the blunt-nosed leopard lizard (*Gambelia sila*), known for its large size, bright nuptial coloration and bipedal sprint bursts [13]. We focus on *G*. *sila* in part because the habitat requirements of the species are generally representative of other upland species of the SJD [7,14]. We use modern and historical land use maps to estimate historical and modern rates of habitat loss and to assess habitat fragmentation. We use historical occurrence records, resurveys, and vegetation measurements to document a changing climatic niche. We also use NASA fallowed area maps to identify 610 km^2^ of fallowed or retired agricultural land with high potential to be restored as habitat. We build habitat suitability models that incorporate known ecophysiological mechanisms that govern species distributions [15–17]. Unlike previous models of habitat suitability for species of the SJD [6,18] (but see [19], S2 Table), our models are appropriate for assessing habitat restoration potential on retired farmland because they properly account for anthropogenic land use. We discuss conservation strategies in light of the potential for habitat restoration and multiple drivers of ongoing and historical habitat loss.

## Methods

### Study system

The San Joaquin Desert (SJD) of California encompasses 28,493 km^2^ including the western and southern two-thirds of the San Joaquin Valley, as well as the Carrizo Plain and Cuyama Valley to the southwest [7]. The SJD is distinguished from the larger San Joaquin Valley by low average precipitation (≤ 279 mm annually), aridic soils, and the presence of a high concentration of co-occurring endemic plant and animal species [8]. The SJD once supported extensive upland habitat composed of alkali sink scrub, saltbush shrub (*Atriplex* spp.), *Ephedra* scrubland, and grassland dominated communities as well as a vast aquatic system of lakes, rivers, marshes, and sloughs fed by rainfall and snowmelt from the Sierra Nevada. Today, most native habitat has been converted to row crops and orchards, remnant upland habitat is heavily impacted by exotic annual grasses and forbs, and wetlands have been drained to support agriculture [20–22]. Thirty-five threatened or endangered species are now confined to isolated patches of habitat in the SJD (S1 Table).

We focus our analyses on habitat of an endemic species of the SJD, *G*. *sila*. *Gambelia sila* were among the first species protected under United States endangered species legislation in 1967 [23] and remain listed as endangered today. Their status is mainly a result of habitat loss and fragmentation, energy development, and non-native vegetation [24]. They use their powerful hind limbs to sprint while evading predators and while catching prey, which consist largely of coleopterans and orthopterans [25]. *Gambelia sila* inhabit relatively flat, sparsely vegetated areas of the SJD including the valley floor, surrounding foothills, and valleys to the southwest [7,8,26].

### Occurrence data

We used 618 geographically unique records of *G*. *sila* occurrence to develop habitat suitability models. We obtained occurrence data from publicly available data portals (e.g., VertNet.org, GBIF.org), the California Natural Diversity Database (CNDDB), correspondence with professional biologists, the literature, and from surveys conducted by the authors of this paper. We corrected for sample bias [27–29] by using all geographically unique vertebrate occurrence records within 50 km of occurrence locations as background or pseudo-absence data (n = 6,285). We did not consider background data within the distribution of the long-nosed leopard lizard (*G*. *wislizenii*) because of evidence of introgression [30,31], potential for competitive exclusion between these congeners [32], and topographic barriers to dispersal. We thinned occurrence and background data to one record per 1-km grid cell to reduce geographic aggregation and spatial sorting bias. We removed areas from model training where occurrence intensity was biased by current land use (e.g., agricultural and urban areas; see methods section on habitat loss and extirpation), allowing our model output to be used as a metric of habitat quality not just on intact habitat, but also as a metric of pre-development habitat quality on lands that have been lost to development.

### Environmental data

We developed habitat suitability models using 11 candidate predictor variables known or hypothesized to be important to *G*. *sila* natural history, demography, and distribution (S3 Table) [13,33]. The 11 variables were composed of continuous metrics of climate (mean annual precipitation [MAP], climatic water deficit [CWD]), thermal physiology (hours of restriction [H_r_], hours of activity [H_a_]), vegetation productivity (normalized difference vegetation index [NDVI], actual evapotranspiration [AET]), soil properties (percentage clay, pH, electrical conductivity), and modeled habitat suitability for a keystone taxon in *G*. *sila* habitat, kangaroo rats (*Dipodomys*), whose precincts and burrows generate high-quality refugia [34,35]. We obtained or derived climate, thermal physiological, and evapotranspiration data from the Basin Characterization Model (270 m resolution, mean values for 1981–2010; [36]). We estimated hours of restriction and hours of activity, the number of hours per day that temperatures are too hot or hot enough for *G*. *sila* activity, by regressing operative environmental temperature [37] data from 12 sites spanning the distribution of *G*. *sila* against maximum daily air temperature data at those sites (S1 Fig) [38,39]. We deployed four models per site in both sun and shade habitats, and thus, 24 models across the species range of two sizes, medium-large (22 × 4 cm) and large PVC (25 × 6 cm), all painted grey, suitable for computing hours of restriction in *G*. *sila*. We derived soil data for surface horizons from the Soil Survey Geographic Database and filled missing areas with values estimated from satellite data [40–42]. We derived average NDVI data from MODIS satellites measurements at 16-d temporal resolution and 250-m spatial resolution over the period 2001–2010. We derived slope from 30-m resolution national elevation dataset (NED) raster grids. We estimated *Dipodomys* habitat suitability as a function of nine predictor variables (S3 Table) using a MaxEnt model parameterized with *Dipodomys* occurrence locations spanning California.

### Model selection and evaluation

We evaluated 236 models, which included all possible, uncorrelated (|*r|* < 0.8) combinations of up to five of the 11 candidate predictor variables. We parameterized models with MaxEnt version 3.3.3k. We turned off hinge and threshold features to reduce overfitting and model complexity. We used the following metrics to evaluate model performance: change in Akaike’s information criterion (ΔAICc), area under the receiver operating characteristic curve (AUC), Boyce Index (BI), and unregularized training gain (Gain). We combined models with > 1% AICc model weight through multi-model averaging [43]. We then used our resulting ensemble habitat suitability model to estimate historical distribution and habitat quality on intact and converted lands and to project potential climate-mediated changes in habitat suitability. We thresholded continuous suitability values into suitable and non-suitable areas using the threshold that maximized the true positive rate and true negative rate.

We used four future climate scenarios to project potential changes in habitat suitability. Climate scenarios were selected to represent a range of potential future conditions, combining two global circulation models with two emission scenarios [44,45]. Circulation models simulate physical processes in the atmosphere, ocean, and land surface. We also assessed current limiting environmental factors for *G*. *sila* across geographic space. We identified the limiting covariate for each grid cell as the covariate providing the greatest increase in habitat suitability if the covariate value was adjusted to its mean value across occurrence locations.

### Habitat loss and extirpation

We estimated the amount of habitat loss from agricultural, urban, and industrial development by overlaying our map of predicted historical distribution onto contemporary and historical land use maps. We obtained historical land use maps for the years 1945, 1960, and 1990 from the Central Valley Historical Mapping Project [20,46]. Land use categories for 1990 were further refined using historical farmland maps from the California Farmland Mapping and Monitoring Program (CFMMP). We obtained contemporary (2015) land use maps from the California Fire Resource and Assessment Program and modified them by hand in accordance with aerial imagery. Estimated habitat loss for intermediate years was interpolated with LOESS regression. We used a statistical relationship between habitat patch size and probability of *G*. *sila* occupancy [47] to estimate per-site probability of occupancy and total amount of habitat loss caused by fragmentation.

We assessed habitat loss at *G*. *sila* historical locations (i.e., extirpation) by reviewing records in the vicinity of areas of development to determine if spatial information (e.g., aerial imagery, land use maps) associated with the records were sufficient to conclude that the habitat had been lost. From 1989–2016, during spring breeding (April to June), we extensively resurveyed two historical record locations on undeveloped habitat at or near the northern limit of the species historical distribution to determine if the species persisted at those sites (S4 Table). We used parametric and nonparametric tests to assess our *a priori* hypothesis that dense herbaceous vegetation was responsible for these extirpations [11,48]. We tallied areas of potential extirpation on intact habitat, where the species was documented historically but has not been seen for decades. We reduced historical (pre-1995) records from intact habitat to one unique occurrence location per 5-km resolution grid cell. We flagged unique historical localities that lacked corresponding recent (1995–present) records within a 5-km radius as areas of potential extirpation.

### Habitat protection and restoration opportunity

We used annual fallowed area maps produced by NASA [49] in conjunction with our historical habitat suitability maps to map the extent of formerly suitable *G*. *sila* habitat that was converted to agriculture and that was continuously out of agricultural production from 2013–2015. We considered these areas to be either retired or to have high potential for permanent retirement from agricultural production. We used logistic regression to assess the probability of 2013–2015 fallowing as a function of CFMMP farmland quality. We used clumping analysis to identify the areas of retired land that, if restored, and in conjunction with existing intact suitable habitat, would constitute continuous areas of suitable habitat ≥ 4.94 km^2^ in size. The 4.94 km^2^ cutoff represented the minimum patch area sufficient for a > 90% probability of local population persistence over the historical era [47]. We used the California Protected Areas Database, the California Conservation Easement Database, and knowledge of additional areas under conservation easement, to identify areas of intact habitat that are currently either protected or not protected from habitat loss. Besides being federally protected, *G*. *sila* is also a California Fully Protected Species, and no loss of habitat is permitted under state law; however, these protections are largely unenforced on agricultural lands (see methods section on habitat protection and restoration opportunity). We used clumping analysis to identify potentially vulnerable areas of unprotected habitat that currently contribute to large areas of intact habitat ≥ 4.94 km^2^ in size.

## Results

### Predicted habitat quality and distribution

The best performing model of current habitat suitability for *G*. *sila* identified, in decreasing order of variable contribution, NDVI, H_r_, slope, percentage clay, and electrical conductivity as the most important drivers of habitat suitability (Table 1). This model had high utility as a predictor of current habitat suitability but was not appropriate for forecasting or hindcasting because of the limited temporal span of the satellite-derived vegetation index, NDVI. To achieve temporal transferability, we performed a second iteration of model selection, limited to the 197 candidate models that did not include NDVI as a predictor variable. In lieu of NDVI, the resulting best-performing models identified hydroclimatic correlates of vegetation productivity (AET, MAP) as the most important predictor variables.

**Table 1:** Performance metrics and variable contribution of top performing habitat suitability models.

	Performance Metric					% Variable Contribution										
A	W	ΔAICc	AUC	BI	Gain	slope	NDVI	AET	CWD	MAP	pH	clay	EC	h_r_	h_a_	dipo
	0.853	0.000	0.931	0.987	1.414	15.5	55.6					5.7	0.5	22.7		
	0.147	3.517	0.930	0.991	1.404	14.5	56.6					5.5		23.3		
	0.000	30.986	0.929	0.989	1.384	17.9	67.2					7.6	0.3		6.9	
	0.000	34.660	0.928	0.995	1.378	17.2	66.2					7.9			6.5	2.2
	0.000	36.930	0.927	0.991	1.370	17.5	67.3					7.9			7.2	
B	W	ΔAICc	AUC	BI	Gain	slope	NDVI	AET	CWD	MAP	pH	clay	EC	h_r_	h_a_	dipo
	0.743	0.000	0.926	0.951	1.368	6.4				86.4	2.5	3.7	0.9			
	0.256	2.130	0.926	0.947	1.366	10.4		77.5			7.2	3.8	1.0			
	0.001	13.406	0.924	0.941	1.350			80.7			11.8	3.3	1.9			2.2
	0.000	17.522	0.923	0.982	1.348	10.5		79.6			4.8		2.5			2.6
	0.000	17.950	0.923	0.809	1.343	7.3				47.1	2.7	4.2			38.7	

Performance metrics are AICc model weight (W), change in Akaike’s information criterion (ΔAICc), area under the receiver operating characteristic curve (AUC), Boyce Index (BI), and unregularized training gain (Gain). Models are ranked in order of increasing ΔAICc. See S3 Table for variable abbreviations and definitions. Blank cells indicate a variable was not included in the model. (A) The top five performing models selected from all 236 candidate models. Satellite derived vegetation productivity (NDVI) is the top predictor of *G*. *sila* distribution, however because NDVI is modified by agriculture it is not an appropriate predictor of historical, paleontological, or potential future distribution. (B) The top five performing models selected from the 197 candidate models that do not include NDVI. The top two performing models from B are the sixth and seventh ranked models (ΔAICc = [40.064, 42.194]) from the full set of 236 candidate models.

The best performing habitat suitability model that is temporally transferable consisted of the weighted average of the top two performing models and incorporated six environmental variables: MAP, AET, slope, percentage clay, electrical conductivity, and pH (Table 1). This ensemble model incorporated 100% of inter-model Akaike weight and also achieved a high AUC score of 0.93. We use this model for all subsequent analyses and figures. Two variables (MAP, AET) indexed hydrology and were related to preference for low herbaceous vegetation density. One variable (slope) is a measure of topography: *G*. *sila* are restricted to relatively flat habitat. Three variables measured soil characteristics: *G*. *sila* appear to have an affinity for lower clay content, more alkaline, and moderately saline soils, perhaps because these characteristics increase friability and reduce vegetation productivity (S2 Fig).

The model threshold that maximized the true positive rate and true negative rate, 0.206, successfully classified 94% of distinct occupancy locations as suitable habitat and classified 81% of background locations (where other species were detected) as unsuitable. These rates suggest that about 6% of real suitable habitat is unaccounted for and that some areas outside of our mapped suitable distribution are perhaps occupied. If detection effort is biased against areas where the model fails to predict suitable habitat, then > 6% of suitable habitat could be missed by the model. Conversely, the thresholded model likely misclassified some of the non-suitable areas as suitable, though without true absence data or repeat visit data, it is not possible to accurately estimate the amount.

**Fig 1:**
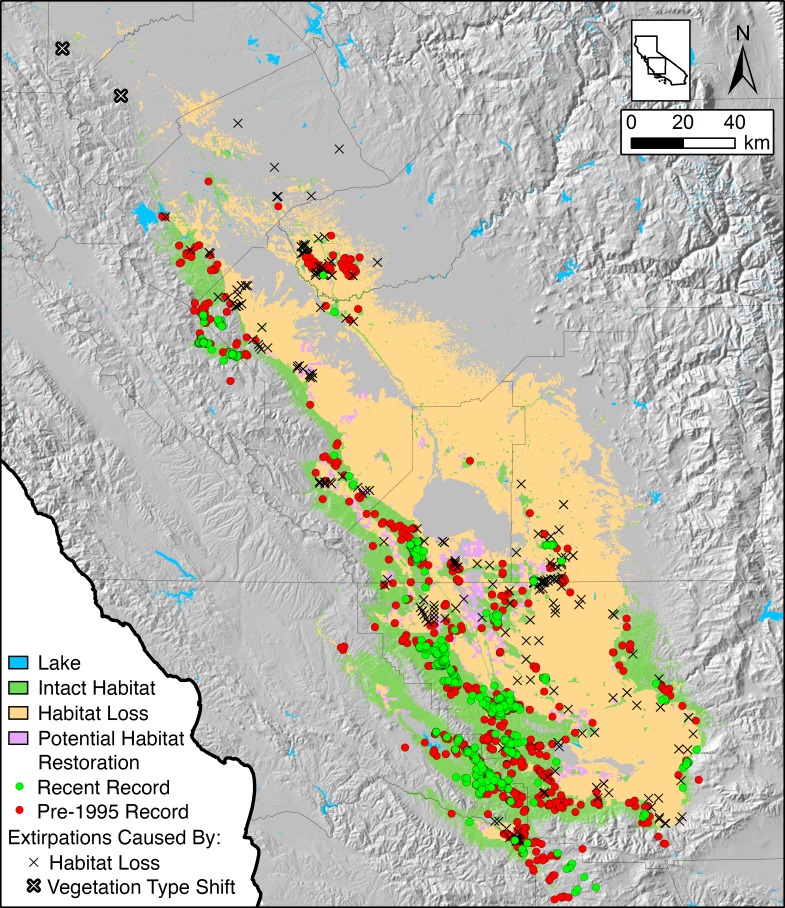
Changing conservation biogeography of *Gambelia sila*. The historical distribution of habitat for *Gambelia sila*, extant sites, extirpated sites, lost habitat, and sites where persistence of *G*. *sila* has not been confirmed since before 1995. Extirpations caused by vegetation type shifts are sites apparently extirpated due to dense exotic vegetation. Areas of potential habitat restoration are sites that were continuously fallow (2013–2015) and, if restored, would constitute a patch of habitat of sufficient size to have a ≥ 90% probability of long-term population persistence. County boundaries are shown in black.

**Fig 2:**
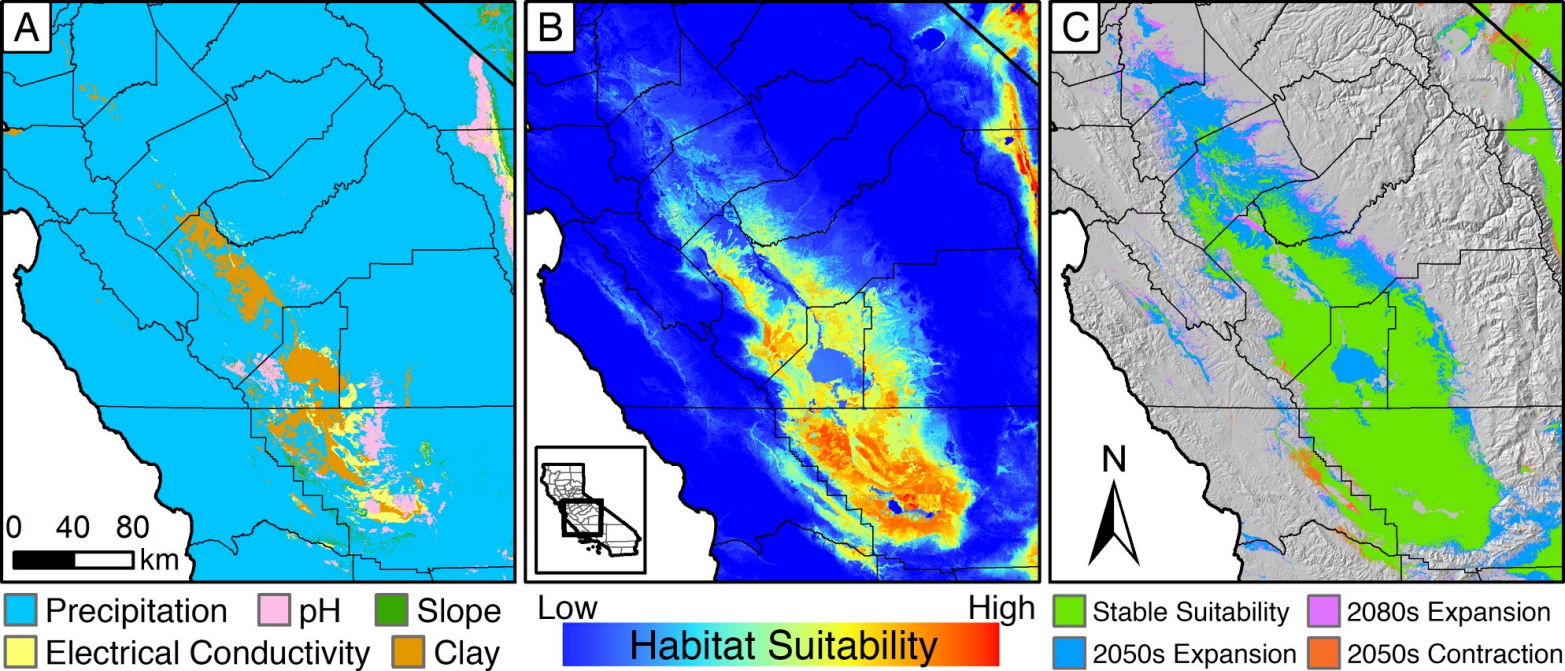
Metrics of habitat suitability for *Gambelia sila*. **(A)** Limiting environmental covariates across geographic space for *Gambelia sila* assessed using the top-performing model of habitat quality and in absence of habitat loss. High precipitation, and resulting high herbaceous vegetation biomass, are the most limiting variables for the lizard (see S3 Table for variable definitions). **(B)** Continuous ensemble habitat suitability over the geographic range of *G*. *sila*. High clay content, acidic, and overly saline soils create pockets of low and non-suitable habitat within the range of the lizard. **(C)** Projected climate-driven change in suitable habitat for *G*. *sila* under a drought scenario (MIROC-ESM, RCP8.5). See S4 Fig for modeled change under four future climate scenarios.

The resulting map of habitat suitability classifies 20,610 km^2^ as historically suitable (Fig 1). The map includes central gaps, which correspond to the historical Tulare, Kern, and Buena Vista lakes, and where high clay content soils reduce suitability today (Fig 2A). Areas of highest predicted habitat quality were found in Kern County, southwestern Tulare County, and on alkaline soils of western Fresno County, including large portions of the Westlands Water District (Fig 2B; S3 Fig). The predicted suitable habitat of *G*. *sila* encompassed occurrence locations for 128 other endangered, threatened, or vulnerable species and contained the majority (≥ 50%) of unique occurrence locations for 40 of these species (CNDDB records).

The projected impact of climate change on *G*. *sila* varied substantially between scenarios of increased versus decreased precipitation (Fig 2C; S4 Fig). Future dry scenarios (MIROC-ESM) resulted in a general trend of northward and peripheral expansion of suitable habitat. Scenarios of increased precipitation (CNRM-CM5) resulted in a general trend of peripheral contraction. Lower greenhouse gas emission scenarios (RCP 4.5) resulted in less change in the geographic boundaries of modeled suitable habitat than higher emission scenarios (RCP 8.5). Modeled habitat suitability declined monotonically in response to increased MAP and AET.

### Habitat loss and extirpation caused by land use

**Fig 3:**
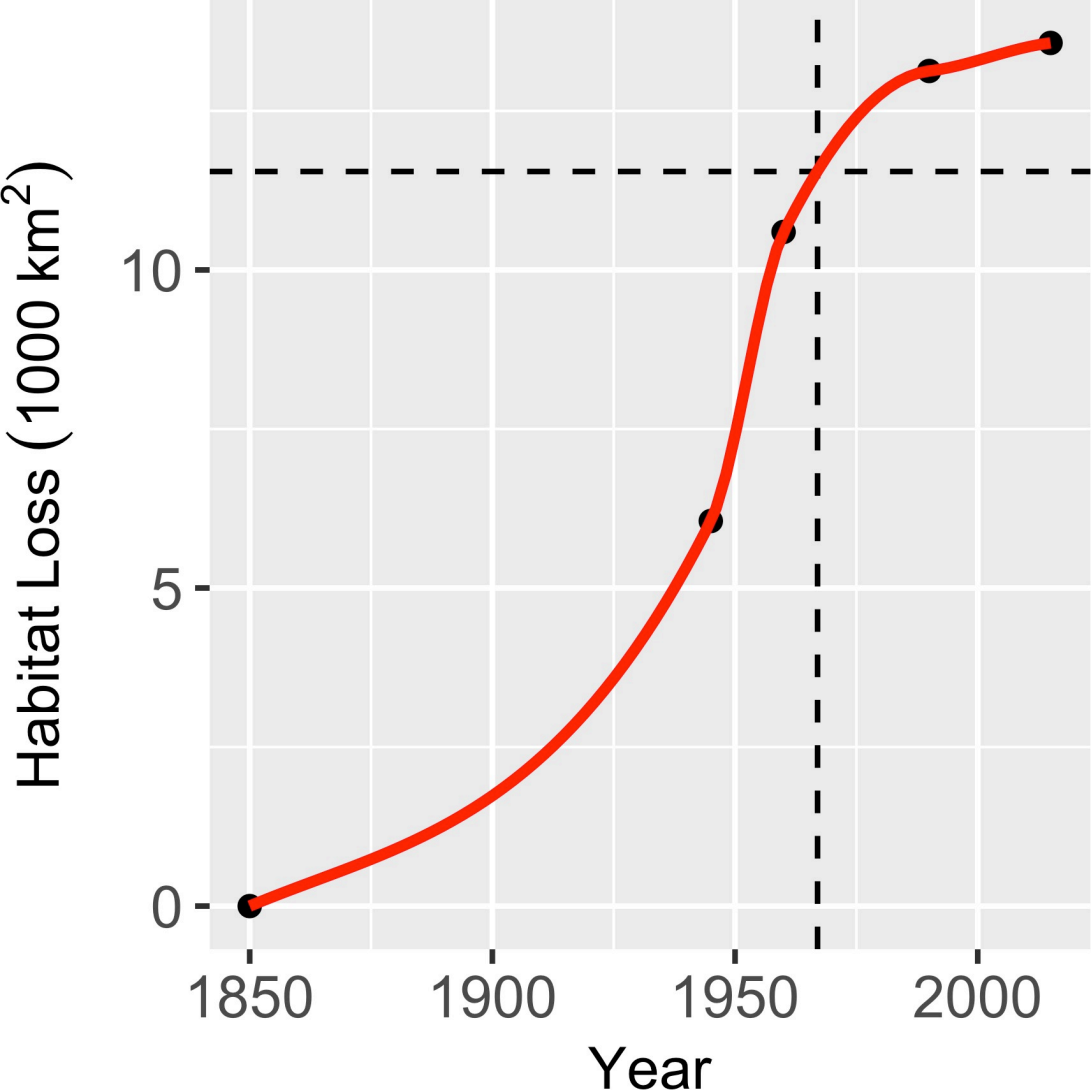
Area of *Gambelia sila* habitat converted to agricultural and urban development over time. Points are derived from the intersection of historical land use maps and predicted historical distribution. The red line is interpolated with LOESS regression. Dashed lines correspond to the year 1967, when the species became federally protected.

We used maps of habitat and land use to estimate that 13,568 km^2^ of *G*. *sila* habitat have been directly lost to agricultural and urban development, comprising 66% of the predicted suitable range of the species. An additional 2.1% (437 km^2^ / 20,610 km^2^) of habitat have been lost to fragmentation caused by development. The rate of habitat loss from agriculture and development appears to have peaked during the 1940s and 1950s, during which time 4,544 km^2^ of habitat were lost (Fig 3). Since 1960, an additional 2,971 km^2^ of habitat were lost to agricultural and urban development. Since protection under the US Endangered Species Preservation Act in 1967 (i.e., forerunner of the 1973 US Endangered Species Act [ESA]), we estimate that 2,021 km^2^ of *G*. *sila* habitat were lost to agricultural and urban development. One hundred and five historical occurrence record locations for *G*. *sila* have been converted to agriculture or other forms of development and were classified as extirpated. At least 45 occurrence record locations where the species was documented after federal protection in 1967 have since been lost to agriculture, urbanization, damning of reservoirs, and other forms of development. Thirty-five of those losses occurred after the species became fully protected under California law in 1970. Many of these lost habitat patches served as corridors connecting larger patches of natural habitat with documented presence of endangered species. At least eight documented occurrence locations were converted to agriculture during the last decade (2007–2016; S5 Table).

### Apparent vegetation-mediated climatic niche contraction

**Fig 4:**
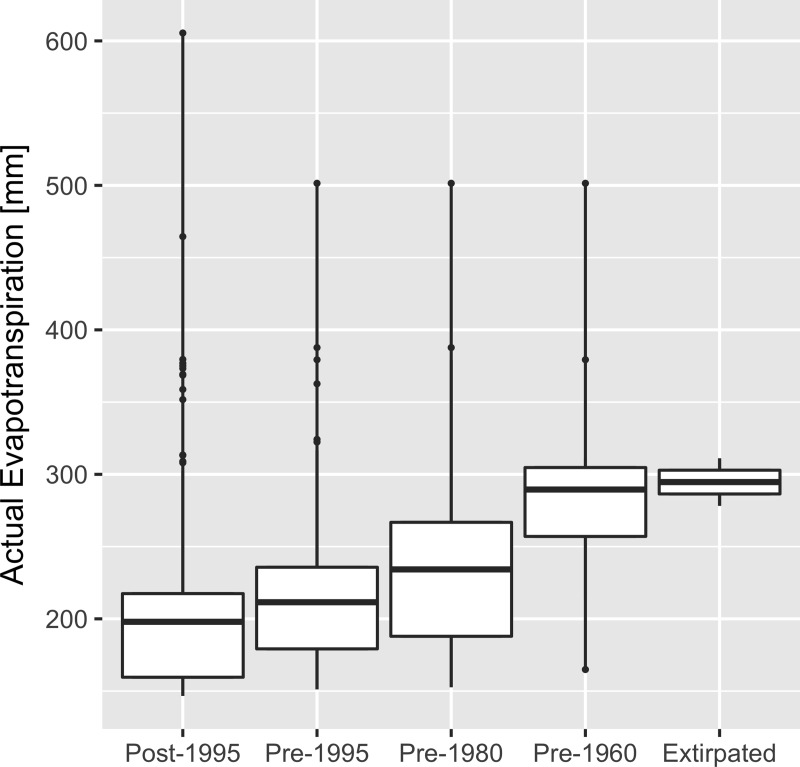
Climatic niche contraction in *Gambelia sila*. Actual evapotranspiration (AET) at *G*. *sila* occurrence locations on undeveloped habitat is higher at sites where *G*. *sila* have not been seen for longer and where *G*. *sila* are now extirpated (Kruskal-Wallis rank sum test, *P* < 0.001). Categories are unique locations (5-km radius) where *G*. *sila* either persist in the modern era (post-1995), were detected only within the given range of years (pre-1995, pre-1980, pre-1960), or where extensive surveys have confirmed extirpation (see Fig 1). Boxes and whiskers depict the median, interquartile, and range.

We did not detect *G*. *sila* after extensive resurvey effort at two historical occurrence locations on apparently intact habitat at, or near, the former northern range limit of the species (Fig 1; S4 Table). These extirpated sites had significantly higher AET (i.e., proxy for vegetation biomass) than 14 extant localities we surveyed for *G*. *sila* in 2014 (Wilcoxon’s signed rank test, *P* < 0.05), and significantly higher AET than 307 recent record locations where the lizard has been recorded since 1995 (Wilcoxon’s signed rank test, *P* < 0.01; Fig 4). The vegetation at northern extirpated sites was dominated by dense exotic grasses and forbs.

AET was significantly higher across all distinct historical (pre-1960) occurrence locations on intact habitat than at modern (1995 and after) distinct occurrence locations (Wilcoxon’s signed rank test, *P* < 0.001; *t*-test, *P* < 0.001; S5 Fig). A total of 103 geographically distinct and pre-modern (pre-1995) record locations on intact habitat lacked modern (1995–present) documentation of local persistence within 5 km of a historical location; we flagged these sites as potentially extirpated. Potentially extirpated sites comprise 49% (103/211) of distinct pre-modern occurrence locations on intact habitat. The most recent observation at 50 of these potentially extirpated historical sites was from before 1980. The most recent observation at 16 of these sites was before 1960. AET was higher at sites where the lizards had not been seen for longer for each of four time-since-last-detection categories (Kruskal-Wallis rank sum test, *P* < 0.001; Fig 4).

### Habitat protection and restoration opportunity

Of the remaining 7,041 km^2^ of *G*. *sila* habitat 1,799 km^2^ (26%) are currently protected as public land or under conservation easements, comprising 8.7% of the precolonial suitable habitat for the species. We identified 5,371 km^2^ of intact and suitable *G*. *sila* habitat that are not currently protected under public ownership or conservation easement. Ninety-one percent (4,903 km^2^) of this intact unprotected habitat contributed to large patches of habitat (> 4.94 km^2^) with high probability (> 90%) of *G*. *sila* population persistence based on patch size. We identified 1,007 km^2^ of potentially retired farmland (continuously fallow 2013–2015) located on formerly suitable habitat for *G*. *sila*, and potentially suitable for habitat restoration and reintroduction. By narrowing our search to prioritize large patches of habitat (> 4.94 km^2^) with high probability (> 90%) of *G*. *sila* population persistence based on patch size, we identified 610 km^2^ of continuously fallow farmland that, with restoration, and in conjunction with existing habitat, would form these large patches of habitat (Fig 1). Farmland quality was a strong predictor of whether land was left fallow during the 2013–2015 drought period (*P* < 0.001), with lesser quality agricultural lands being far more likely to be fallow during this period.

## Discussion

### Conservation trajectory

Despite the presence of dozens of threatened and endangered species, loss of natural habitat continues in the SJD. Over the past half century, habitat destruction in the SJD has slowed but it has not halted or reversed (Fig 3). The estimated amount of *G*. *sila* habitat lost since the species became protected is greater than the total amount of habitat currently protected through public ownership and conservation easement. Unmitigated habitat loss to agricultural and other land conversion continues on large parcels of habitat, including areas with documented *G*. *sila* occurrence, areas adjacent to protected lands, and areas that formerly served as corridors connecting large patches of habitat (S5 Table). These trends appear to be generalizable to other upland endangered species of the SJD.

Where habitat remains undeveloped, invasion of exotic annual grasses and forbs appears to be responsible for peripheral range contraction from the mesic margins of the distribution of *G*. *sila*. Before European colonization of California, native habitat provided areas of relatively bare soil [21], important for lizard locomotion while hunting and evading predators, and for basking [13]. Today, widespread invasion by exotic annual grasses and forbs has resulted in dense thatch that precludes these behaviors, and leads to demographic decline [10,11], particularly in peripheral portions of the species range where higher precipitation adds to herbaceous productivity. Though many invasive grasses and forbs that affect *G*. *sila* were first introduced to California more than a century ago, the patterns we observe in occurrence data suggest that vegetation-mediated range contraction of *G*. *sila* may be still unfolding (Fig 4). The full effects of biological invasions are mediated by stochastic processes and can take millennia to unfold [50,51]. Anthropogenic nitrogen deposition has likely exacerbated the impacts of exotic grasses and forbs on SJD endemic species, and its impacts are worthy of further investigation [52]. Grazing by livestock and native kangaroo rats can reduce thatch and mitigate the impact of invasive grasses and forbs [35,48]; however, even in areas under active management (e.g., vegetation restoration, grazing to thin excess herbaceous growth, etc.), such as at Allensworth Ecological Reserve, Pleasant Valley Ecological Reserve, and Pixley National Wildlife Refuge, *G*. *sila* populations have declined precipitously since the 1990s [24].

The high proportion (49%) of historical occurrence locations on intact habitat where *G*. *sila* have not been documented for over two decades is of great concern. Many of these sites may have suffered extirpation as a result invasion by exotic annual grasses and forbs. Over 25 years ago, Germano and Williams [26] identified a range-wide status survey as a top priority for *G*. *sila* recovery, noting that “no status survey has ever been conducted, even though the species was first federally listed in 1967.” We echo the importance of conducting this type of range-wide survey. A status survey is necessary to fully evaluate the conservation status and potential future recovery of the species. Resurveying old occurrence locations may also aid in resolving uncertainty in how species will respond to climate change (S1 Text). Few reports from previous surveys have recorded where *G*. *sila* were not detected (but see [47]). Documenting such information would enable ecologists to shift from a modeling framework based on presence data only to a more robust occupancy modeling framework and improve the capacity for species management and conservation.

Three previous studies used non-quantitative methods to estimate the proportion of *G*. *sila* habitat lost to development. They estimated that between 80–94% of habitat had been lost [10,26,53]. Based on our analysis of habitat lost to agriculture, development, and fragmentation (68% of habitat), discovery of apparent vegetation and climate-mediated extirpations and range contraction, the large proportion of sites where *G*. *sila* have not been seen for decades, and other sources of unquantified habitat loss and degradation, we conclude that these previous estimates may reasonably bracket the proportion of habitat loss and range contraction experienced by the species. Other unquantified sources of habitat loss and degradation include off-road vehicle use, petrochemical extraction, solar infrastructure, aerial application of insecticides, and atmospheric nitrogen deposition [24,52].

### Habitat protection, restoration, and reintroduction priorities

In the midst of the downward trend in intact habitat in the SJD, a trend toward retirement of marginal farmland has also emerged [49]. Much farmland in the western SJD is of marginal quality and suffers from salinization due to irrigation of saline soils with low permeability clay layers [54], making irrigated agriculture unsustainable [55]. Climate change in the SJD is also contributing to reduced water availability and increased evaporation [56–58]. The trend toward fallowing and retirement of farmland is projected to continue as climate change exacerbates drought stress and basins come into compliance with California’s Sustainable Groundwater Management Act [9].

We identified 610 km^2^ of farmland with strong potential for habitat restoration. These lands were continuously fallow for three years of the California megadrought (2013–2015) and would contribute to sufficiently large patches of habitat for a high probability of *G*. *sila* population persistence. Because the drivers of which lands are retired in response to reduced water availability are likely to be constant over time (i.e. farmland soil quality, water rights) we believe that these lands can serve as a preview of some of the areas that are likely to be retired over the coming decades. With more than 2,000 km^2^ of SJD farmland projected to be retired in the next 30 years as basins adapt to reduced water availability, habitat restoration could represent an important contribution toward the recovery of dozens of threatened and endangered species. Restoration is attractive because it potentially reverses the trend of habitat loss as opposed to merely slowing decline. Nevertheless intact habitat tends to be superior to restored habitat [59]. Efforts toward restoration should not supplant, but rather should supplement traditional methods of habitat protection and management. Both approaches may be used in concert to conserve a diverse portfolio of sufficiently large patches of habitat.

The prospect of restoring land that is no longer cost-effective for agriculture may represent an efficient means of habitat conservation; however, more knowledge and experimentation is needed to understand the timeline and parameters that influence habitat suitability for threatened and endangered species on such lands [60]. Currently, only one study has evaluated restoration on retired farmland in the SJD [61]. The study evaluated upland restoration treatments ranging from the “do nothing approach” of simply letting natural processes carry out on their own, to more intensive treatments, including various combinations of sowing native seeds, burning, weed management, irrigation, and microtopographic grading. Among other findings, Laymon et al. [61] found the number of years that sites were fallow was positively correlated with native plant cover. Elsewhere, we have observed that *G*. *sila* and other endangered species have recolonized dryland farmland that has been retired for decades in the absence of any restoration interventions (S6 Table). Given enough time, and proper conditions, simply retiring land may be sufficient for some aspects of habitat recovery. Low-cost, high-reward interventions that could expedite recovery might include translocating native ecosystem engineers such as Heermann’s kangaroo rats (*Dipodomys heermanni*), re-establishing native shrubs, microtopographic grading, and a combination of targeted grazing by livestock and burning to control weeds [11,35,48,61]. In addition to treatments mentioned above, translocations of key vertebrate and invertebrate species and inoculation of soil microorganism may be beneficial when local sources are not present [62]. Restoration efforts should serve as experiments for evaluating the context dependent efficacy of various treatments. If success can be demonstrated, restoration could serve in tandem with protection of undisturbed lands as an effective strategy for recovery of threatened and endangered species.

We encourage consideration of the following factors in prioritizing land for protection and restoration. First, sandy and alkaline soils appear to be ideal for conservation in the SJD; they support less growth by exotic grasses and forbs, they are associated with occurrence of *G*. *sila* on intact habitat (S2 Fig), and they have higher native plant cover following habitat restoration on farmland [61]. Second, potential linkages between existing patches of protected habitat may be especially valuable and should be prioritized [63]. Our maps reveal several such potential linkages including both on unprotected intact habitat and on farmland with strong potential for retirement and restoration. Third, many of the areas with high potential for permanent retirement encompass or are proximate to historical occurrence records of *G*. *sila*, providing additional evidence that these areas once served as habitat and have potential to again serve as habitat. These include areas that are not adjacent to intact habitat and where translocation may be necessary to re-establish populations. Finally, a prudent strategy for conserving endangered species in the face of uncertainty is to maintain a diverse portfolio of genetic lineages on climatically and environmentally differentiated habitats [64]. Recent analysis of *G*. *sila* genomic and mitochondrial datasets [31] identify six regional groups that generally align with recovery areas designated by the U.S. Fish and Wildlife Service [24]. Conservationist should prioritize habitat protection for the clades that are underrepresented by current habitat protections.

## Supporting information

S1 Text Discussion of potential impact of climate change. (PDF)

S1 Table Threatened, endangered, extinct, and extirpated species of the San Joaquin Desert. List includes 42 species with occurrence records that fall within the boundary of the San Joaquin Desert (*sensu* Germano et al., 2011). SSC indicates a California species of special concern.
(XLSX)

S2 Table Biases and critiques of previous species distribution models for San Joaquin Desert species. (XLSX)

S3 Table Information on 11 candidate predictor variables evaluated for their strength in determining habitat quality and distribution. (XLSX)

S4 Table Summary of resurvey effort for two apparently extirpated historical record locations at or near the historical northern range margin of *Gambelia sila*. (XLSX)

S5 Table Locations of some recent *Gambelia sila* habitat destruction. This list is by no means comprehensive. It is a partial list of locations where the authors and collaborators have observed habitat loss in the course of other work duties. Examining historical aerial imagery in the vicinity of many of these disturbances reveals additional instances of habitat loss that are not included in this table. Year and acreage of disturbances may represent multi-year habitat erosion processes.
(XLSX)

S6 Table Locations of *Gambelia sila* occurrence observed on retired agricultural lands. Scars from former ploughing are clearly visible on aerial imagery of these sites.
(XLSX)

S1 Fig **Hours of restriction during the breeding season (left) and hours of activity during the active season (right).** Hours of restriction are average number of hours per day during the breeding season (AMJJ) that operative environmental temperatures are too hot for *Gambelia sila* to be active above ground. Hours of activity are number of hours per day during the active season (AMJJASO) that operative environmental temperatures are hot enough for *G*. *sila* to be active [17]. *Gambelia sila* occurrence locations are shown in black. Values are derived from temperatures from 1981–2010.
(TIFF)

S2 Fig Density plots for 11 candidate predictor variables. Shown are *Gambelia sila* occurrence locations and background sampling locations used for parameterizing our models. Occurrence data was thinned to one record per 1-km grid cell. Old locations on developed habitat were not included.
(TIFF)

S3 Fig Habitat suitability in the Westlands Water District peaks on alkaline soils located in the western portions of the district. Under a settlement negotiated with the federal government at least 405 km^2^ of farmland in Westlands Water District will be permanently retired, including 70–210 km^2^ of formerly suitable habitat for *Gambelia sila*. The thick border is Westlands Water District boundary. Thin borders are county boundaries. For information on the settlement between the federal government and Westlands Water District see https://wwd.ca.gov/resource-management/drainage-settlement-documents/.
(TIFF)

S4 Fig Modeled change in habitat suitability over time for four future climate scenarios. Climate scenarios were selected to represent a range of potential future conditions, combining two global circulation models with two emission scenarios. The global circulation models predict either a relatively hot and dry future (MIROC-ESM) or a relatively warm and wet future (CNRM-CM5). The emission scenarios represent either relatively high (RCP 8.5) or relatively low (RCP 4.5) emission trajectories. Decreased precipitation leads to a predominant trend of northward expansion in the MIROC-ESM scenarios. Conversely, increased precipitation leads to peripheral contraction in the CNRM-CM5 scenarios.
(TIFF)

S5 Fig Change in climatic niche of *Gambelia sila* from the historical era to modern era with respect to actual evapotranspiration (AET). The distribution of all distinct *G*. *sila* record locations on intact habitat has shifted toward sites with lower AET from the historical (pre-1960) to modern (1995 or after) periods.
(TIFF)

S6 Fig Comparison of realized climatic niches for *Gambelia sila* and all three species in the genus *Gambelia*. Other members of the genus occupy hotter and drier environments than are available to *G*. *sila* in the San Joaquin Desert (see also S2 Fig). Occurrence data were thinned to one record per 30-arcsecond climate grid cell. Climate data were extracted from 30-arcsecond resolution WorldClim surfaces for the period 1960–1990 instead of from the Basin Characterization Model (used in all other analyses; see text) because occurrence data extends beyond the domain of the later.
(TIFF)

S1 File Ensemble habitat suitability surfaces generated for this study. Zipped file includes GeoTIFF files representing continuous and binary historical habitat suitability for *Gambelia sila* (see text).
(ZIP)
