# Supplementary material for: Habitat restoration opportunities, climatic niche contraction, and conservation biogeography in California's San Joaquin Desert
Source: PLoS One. 2019 Jan 15;14(1):e0210766. doi: 10.1371/journal.pone.0210766 (PMC6333358; doi:10.1371/journal.pone.0210766)
Supplement: S1 Text — (PDF) [file pone.0210766.s001.pdf]

Supplementary online text for:

Habitat restoration opportunities, climatic niche contraction, and conservation biogeography in California's San Joaquin Desert

Joseph A E Stewart, H Scott Butterfield, Jonathan Q Richmond, David J Germano, Michael F Westphal, Erin N Tennant, Barry Sinervo

S1 Text. Discussion of potential impact of climate change.

Much uncertainty remains in how blunt-nosed leopard lizards (*Gambelia sila*) will respond to climate change. Given uncertainty in the impacts of climate change, the ideal conservation strategy may be functionally equivalent to the ideal conservation strategy in the absence of climate change: managers should maintain a diverse portfolio of genetic lineages on environmentally diverse habitats [1].

On the mesic margin of the species' distribution, historical and modern distributional limits appear to be governed by herbaceous vegetation productivity (i.e. AET, S2 Fig). This limit to the species' climatic niche is supported by multiple lines of evidence: demographic decline in response to high precipitation years with high herbaceous biomass [2], observations of *G. sila* having difficulty moving through dense thatch, the apparent climatic niche contraction mediated by invasive species we document in this paper (see results section on apparent vegetation-mediated climatic niche contraction), and geographic patterns in occurrence data. Accordingly, our distribution models are sensitive to changes in precipitation and evapotranspiration, with scenarios of decreased future precipitation resulting in projections of peripheral range expansion and scenarios of increased future precipitation resulting in projections of peripheral range contraction (S4 Fig).

While the current distribution of *G. sila* is limited by excess water availability, it does not appear that its distribution is currently limited by hot or dry limits to its climatic niche. The species currently occupies the hottest and driest portions of its geographic range in the San Joaquin Desert (S2 Fig). Though authors of this paper documented temporary cessation of reproduction in response to extreme drought conditions and water year precipitation below 92 mm [3,4], no instances of extirpation or range limitation appear to be associated with hot or dry conditions. Population viability analyses may be necessary to assess whether potential drought scenarios could pose a risk for *G. sila*. Further, other members of the genus *Gambelia* occur in hotter and drier environments than are occupied by *G. sila* (S6 Fig), suggesting that *G. sila* could possess capacity to tolerate similar conditions.

We urge caution in interpreting our projections of changes in habitat suitability under potential climate change scenarios (Fig 2C and S4 Fig). The projections we present were selected to represent approximate bounds of the range of projected change in precipitation represented in CMIP5 for California. Most future climate scenarios project less change in mean annual precipitation in California than the scenarios presented, with end-century ensemble means approximating no change in mean annual precipitation [5]. Additionally, the model does not account for projected increases in interannual precipitation variability [6], which could negatively impact *G. sila* throughout its range [2,3]. Developing models that account for the response of *G. sila* to these components of climate change may be possible with sufficient demographic data. Populations residing on habitat that features edaphic and topographic diversity may be more robust to forecasted increases in interannual precipitation variability.

References

1. Lawler JJ. Climate change adaptation strategies for resource management and conservation planning. Ann N Y Acad Sci. 2009;1162:79–98. doi:10.1111/j.1749-6632.2009.04147.x
2. Germano DJ, Williams DF. Population ecology of blunt-nosed leopard lizards in high elevation foothill habitat. J Herpetol. 2005;39:1–18.
3. Westphal MF, Stewart JAE, Tennant EN, Butterfield HS, Sinervo B. Contemporary drought and future effects of climate change on the endangered blunt-nosed leopard lizard, *Gambelia sila*. PLoS One. 2016;11:e0154838. doi:10.1371/journal.pone.0154838
4. Germano DJ, Williams DF, Tordoff W. Effect of drought on blunt-nosed leopard lizards (*Gambelia sila*). Northwest Nat. 1994;75:11–19.
5. Thorne JH, Boynton RM, Holguin AJ, Stewart JAE, Bjorkman J. A climate change vulnerability assessment of California's terrestrial vegetation. Sacramento, CA; 2016. doi:10.1371/journal.pone.0029507
6. Swain DL, Langenbrunner B, Neelin JD, Hall A. Increasing precipitation volatility in twenty-first-century California. Nat Clim Chang. Springer US; 2018;8:427–433. doi:10.1038/s41558-018-0140-y
