# Supplementary figures and images for: Habitat restoration opportunities, climatic niche contraction, and conservation biogeography in California's San Joaquin Desert
Source: PLoS One. 2019 Jan 15;14(1):e0210766. doi: 10.1371/journal.pone.0210766 (PMC6333358; doi:10.1371/journal.pone.0210766)

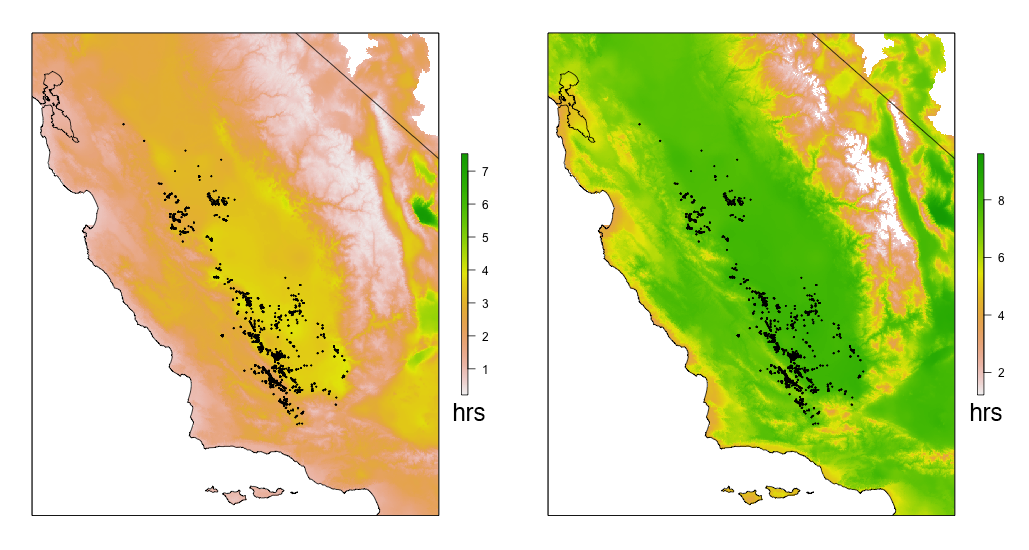

Supplement: S1 Fig — Hours of restriction during the breeding season (left) and hours of activity during the active season (right). Hours of restriction are average number of hours per day during the breeding season (AMJJ) that operative environmental temperatures are too hot for Gambelia sila to be active above ground. Hours of activity are number of hours per day during the active season (AMJJASO) that operative environmental temperatures are hot enough for G. sila to be active [17]. Gambelia sila occurrence locations are shown in black. Values are derived from temperatures from 1981–2010. (TIFF) [file pone.0210766.s008.tiff]

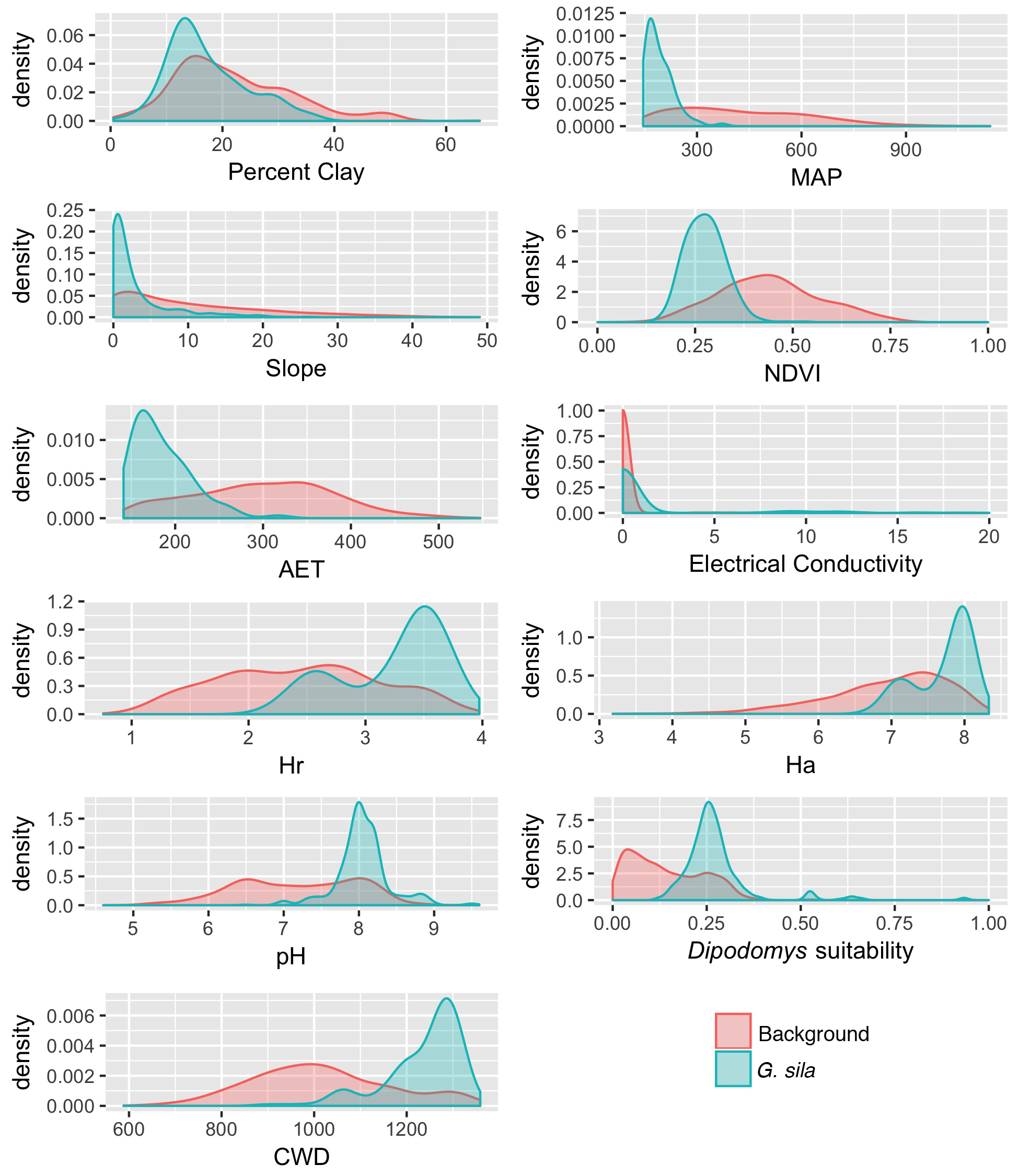

Supplement: S2 Fig — Shown are Gambelia sila occurrence locations and background sampling locations used for parameterizing our models. Occurrence data was thinned to one record per 1-km grid cell. Old locations on developed habitat were not included. (TIFF) [file pone.0210766.s009.tiff]

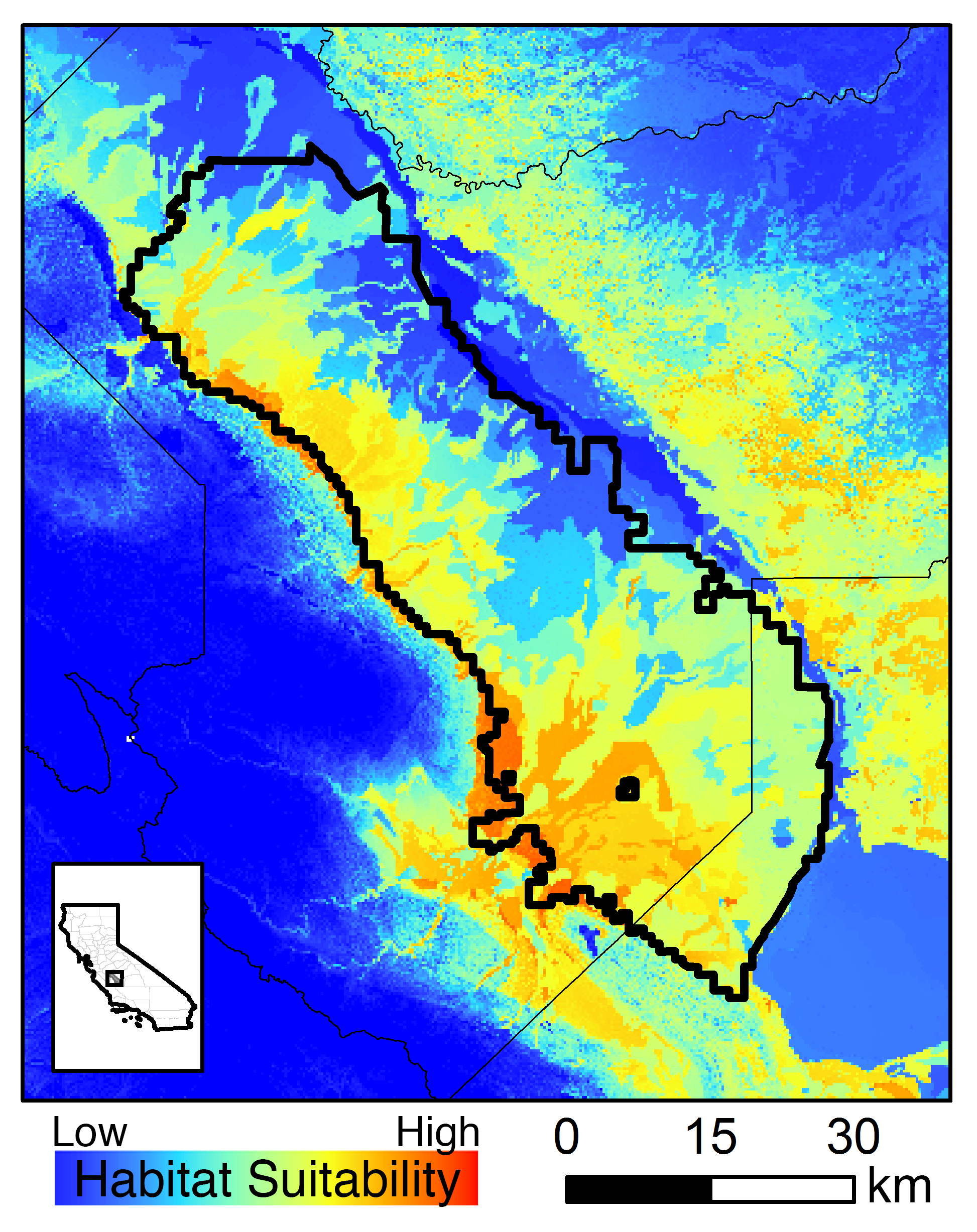

Supplement: S3 Fig — Under a settlement negotiated with the federal government at least 405 km2 of farmland in Westlands Water District will be permanently retired, including 70–210 km2 of formerly suitable habitat for Gambelia sila. The thick border is Westlands Water District boundary. Thin borders are county boundaries. For information on the settlement between the federal government and Westlands Water District see https://wwd.ca.gov/resource-management/drainage-settlement-documents/. (TIFF) [file pone.0210766.s010.tiff]

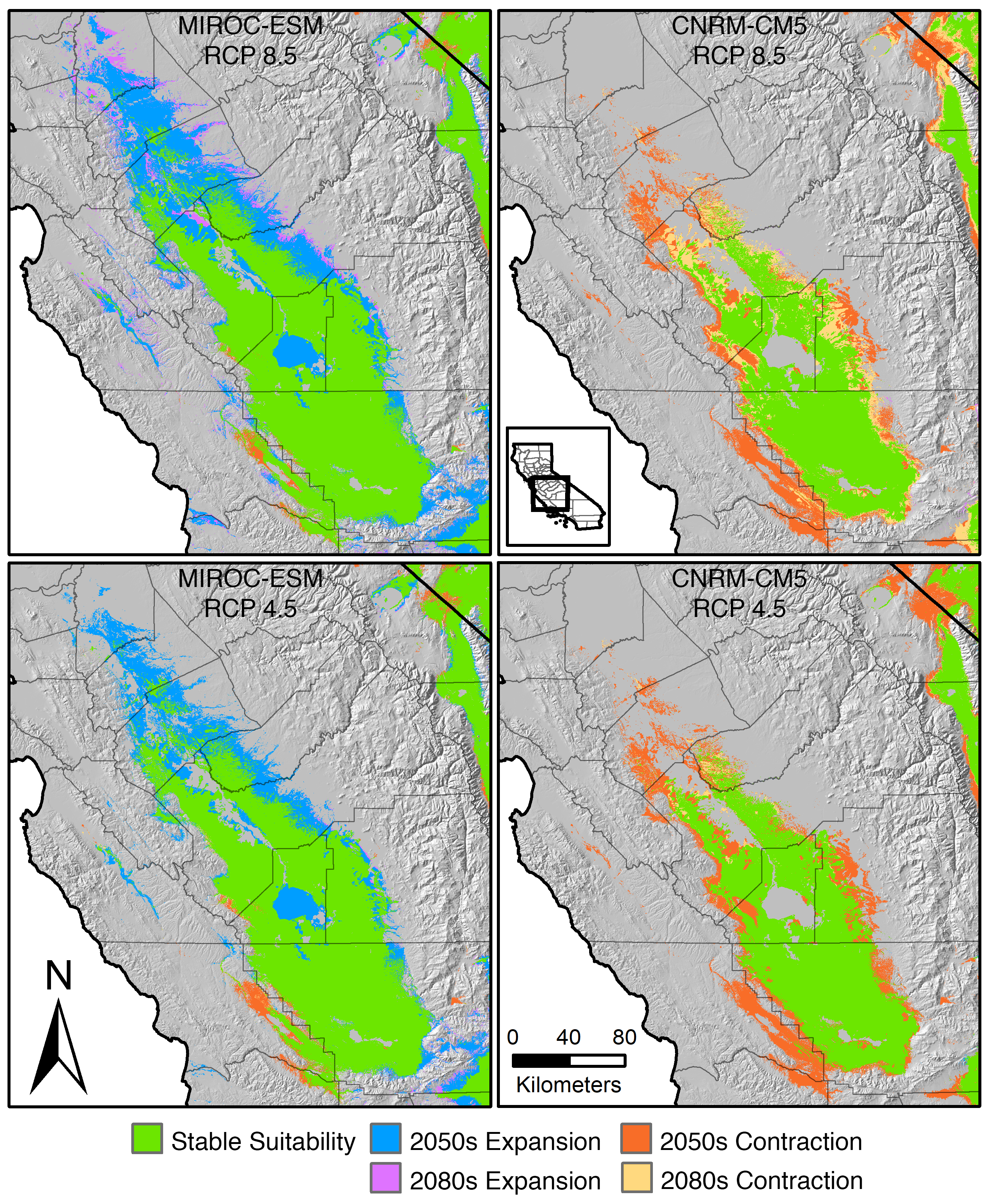

Supplement: S4 Fig — Climate scenarios were selected to represent a range of potential future conditions, combining two global circulation models with two emission scenarios. The global circulation models predict either a relatively hot and dry future (MIROC-ESM) or a relatively warm and wet future (CNRM-CM5). The emission scenarios represent either relatively high (RCP 8.5) or relatively low (RCP 4.5) emission trajectories. Decreased precipitation leads to a predominant trend of northward expansion in the MIROC-ESM scenarios. Conversely, increased precipitation leads to peripheral contraction in the CNRM-CM5 scenarios. (TIFF) [file pone.0210766.s011.tiff]

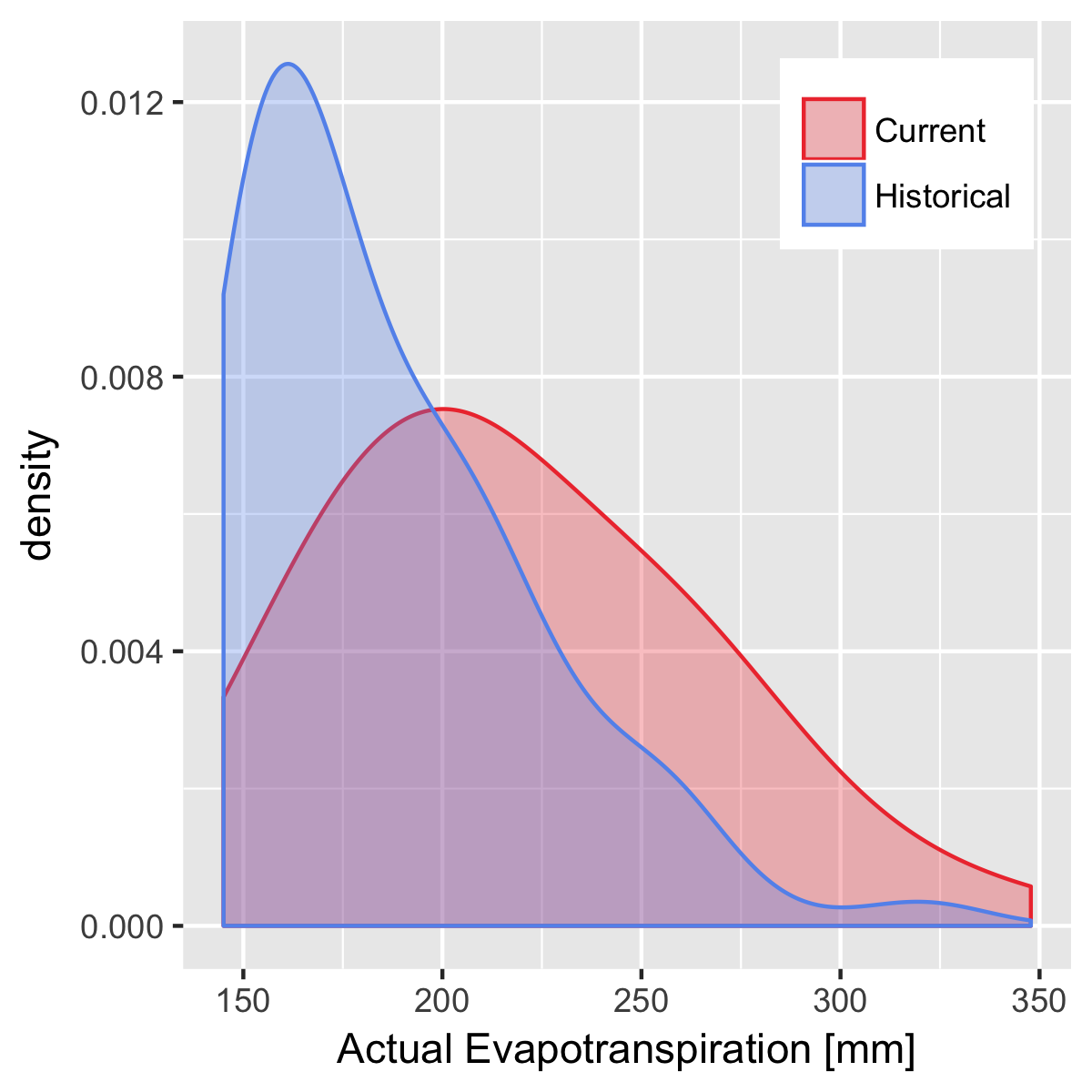

Supplement: S5 Fig — The distribution of all distinct G. sila record locations on intact habitat has shifted toward sites with lower AET from the historical (pre-1960) to modern (1995 or after) periods. (TIFF) [file pone.0210766.s012.tiff]

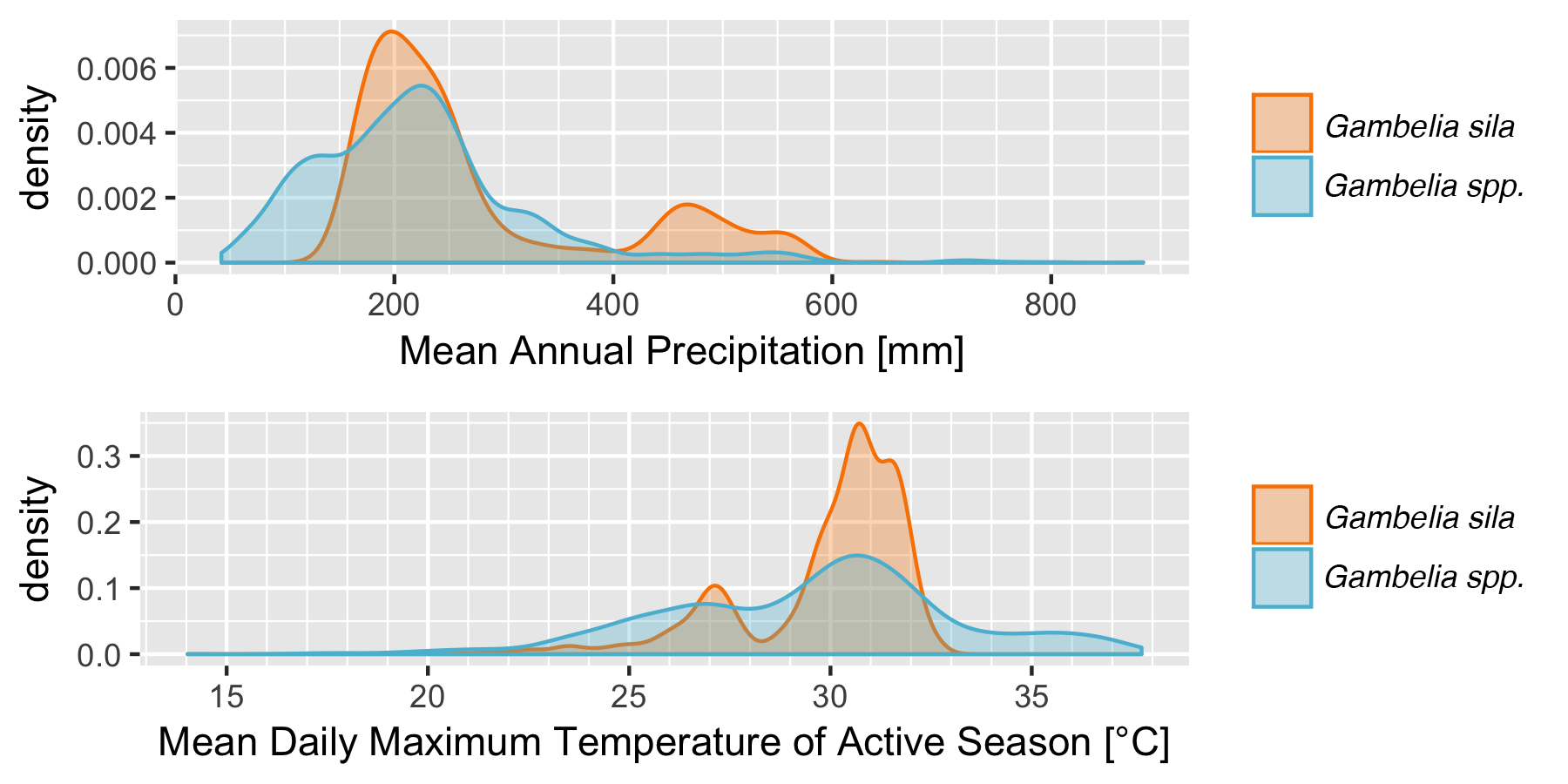

Supplement: S6 Fig — Other members of the genus occupy hotter and drier environments than are available to G. sila in the San Joaquin Desert (see also S2 Fig). Occurrence data were thinned to one record per 30-arcsecond climate grid cell. Climate data were extracted from 30-arcsecond resolution WorldClim surfaces for the period 1960–1990 instead of from the Basin Characterization Model (used in all other analyses; see text) because occurrence data extends beyond the domain of the later. (TIFF) [file pone.0210766.s013.tiff]
